# Supplementary material for: The myosin X motor is optimized for movement on actin bundles
Source: Nat Commun. 2016 Sep 1;7:12456. doi: 10.1038/ncomms12456 (PMC5025751; doi:10.1038/ncomms12456)
Supplement: Supplementary Information — Supplementary Figures 1-13, Supplementary Tables 1 & 2, Supplementary Note, Supplementary References [file ncomms12456-s1.pdf]

SUPPLEMENTARY FIGURES

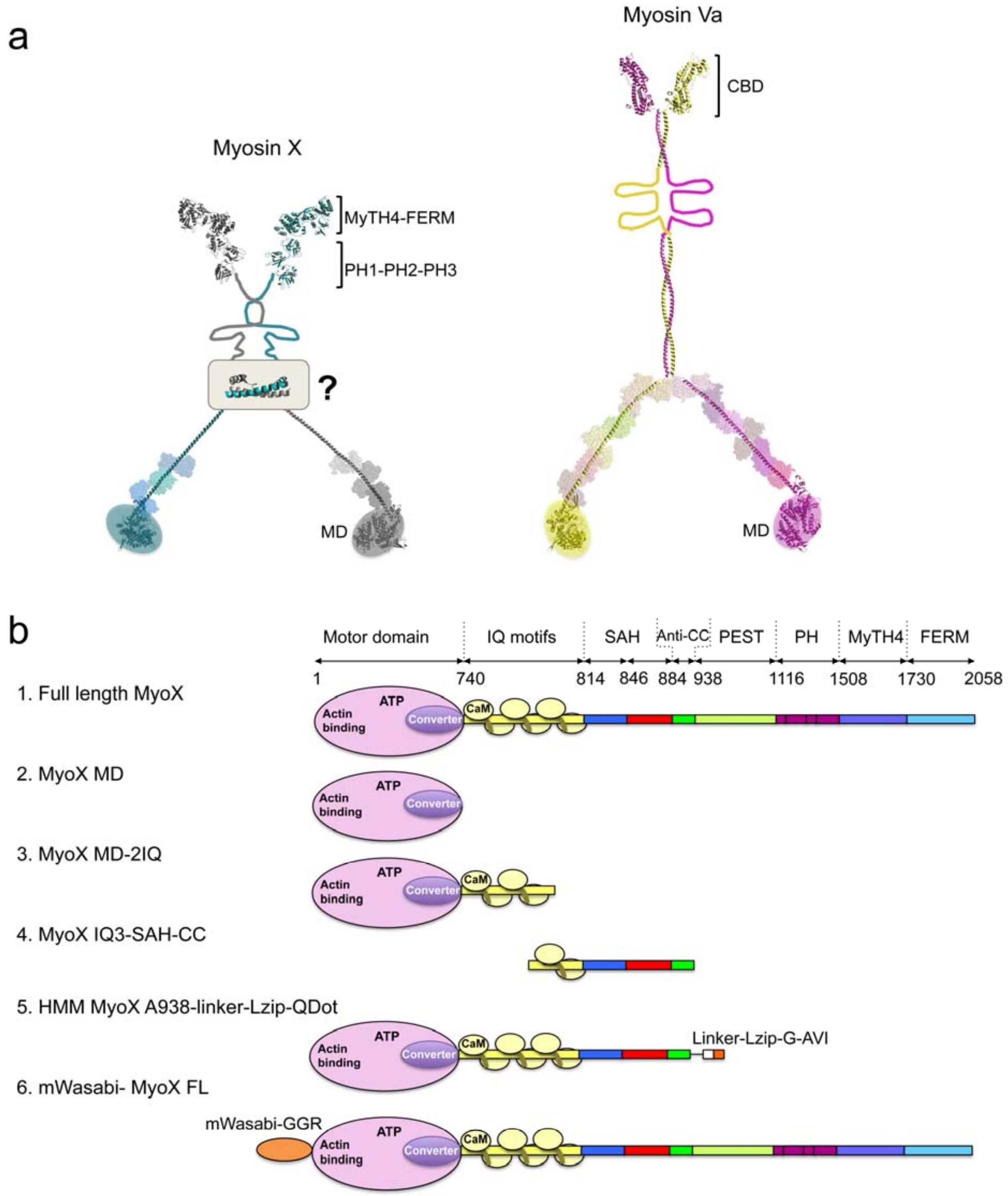

Supplementary Figure 1. Myosin X architecture and constructs. **(a)** Comparison of myosin X and myosin V design for lever arm, dimerisation and globular tail domains. Differences in these features underline large differences in motility and cellular function for these myosins. The myosin X tail contains pleckstrin homology (PH) domains and a MyTH4-FERM domain. These C-terminal domains can potentially be the binding sites for molecules such as phosphatidylinositol 3,4,5-triphosphate, microtubules, and integrins and thus target myosin X within a cell[1-4]. **(b)** Constructs of Myosin X used in this study.

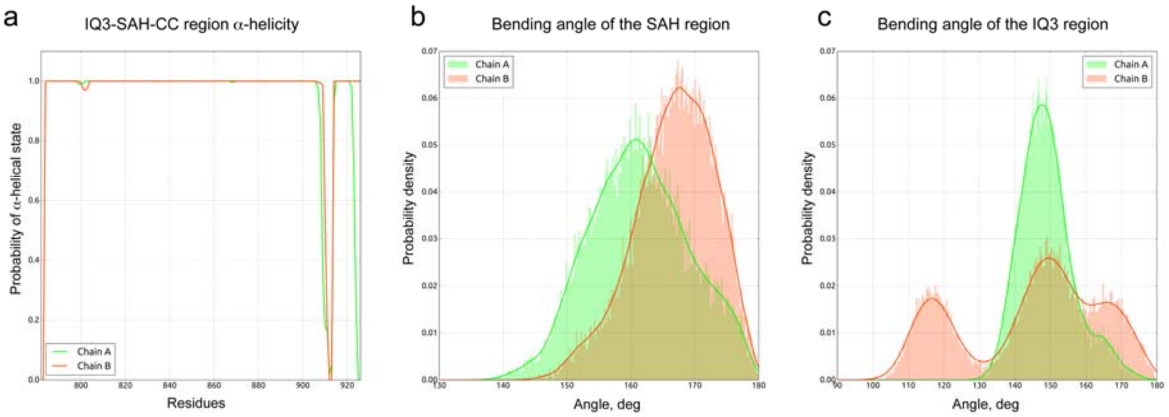

Supplementary Figure 2. Flexibility in the lever arm and dimerization region. (a)-(c) Flexibility and secondary structure content of the myosin X IQ3-SAH-CC region studied by molecular dynamics simulations. **(a)** Secondary structure of the SAH-CC region during the simulation. The SAH region remains fully helical all along the simulation. The unfolded segment around residues 910-915 corresponds to the flexible linker within the two helices of the dimerization region. **(b)** Statistical distribution of the bending angle of the SAH region, defined as the angle between the α-carbons of residues 800-852-903. Although both chains of the dimer seem to favor near-straight conformations (average bending angle 161.4°, standard deviation 7.7° for chain A; average bending angle 166.8°, standard deviation 6.3° for chain B), bent conformations (up to ~140° for chain A) are observed punctually. **(c)** Statistical distribution of the bending angle at the junction between the IQ3 and SAH regions, defined as the angle between the α-carbons of residues 783-800-832.

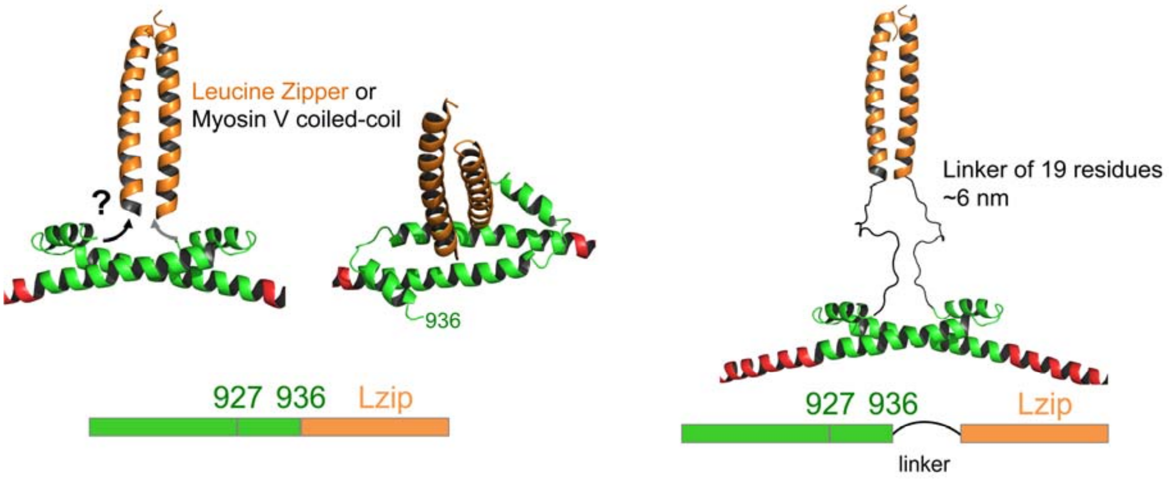

Supplementary Figure 3. Designs of Myosin X-Leucine zipper chimeras. (Left) Cartoon representation of Myosin X HMM construct from previous work[16-18] where the Leucine-zipper (or Myosin V) coiled-coil is directly connected to the residue 936 of Myosin X. (Right) Our HMM chimera in cartoon mode containing a linker of 19 residues (SEGGSGGSGGSGGSAASAA) between the residue 936 of Myosin X and the leucine zipper.

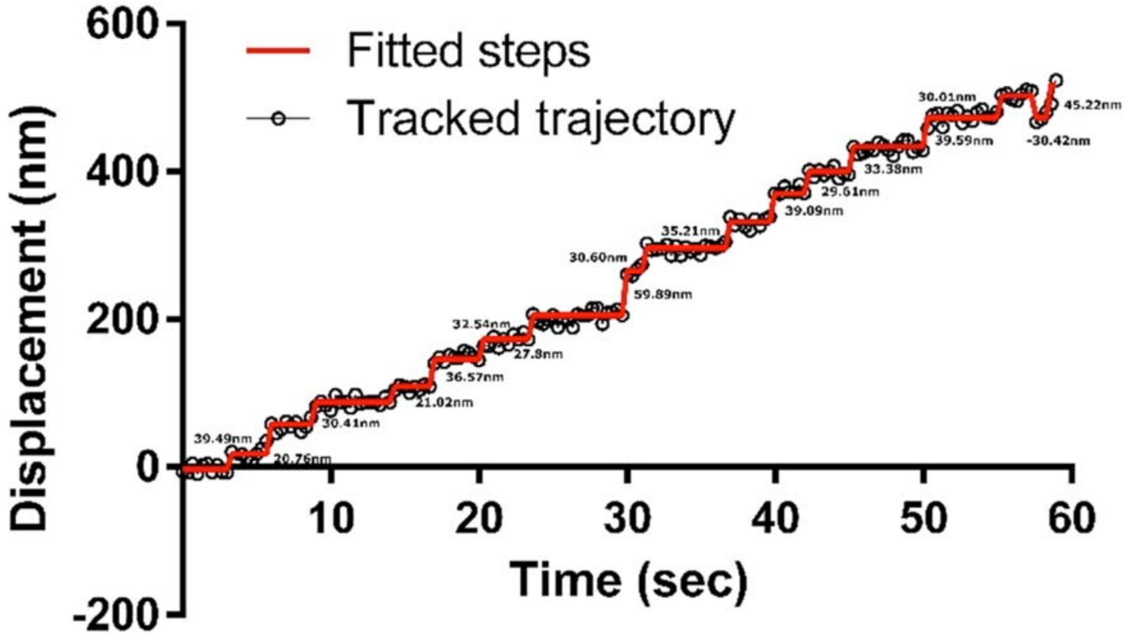

Supplementary Figure 4. Myosin X full length processive stepping on fascin-bundled F-actin filament. Myosin FL dimerized and move processively on F-actin filaments and fascin bundled F-actin filaments. This shows an example of a tracked trajectory of full-length myosin X motility on fascin-bundled F-actin filament under low MgATP condition (0.4µm MgATP) in the motility buffer.

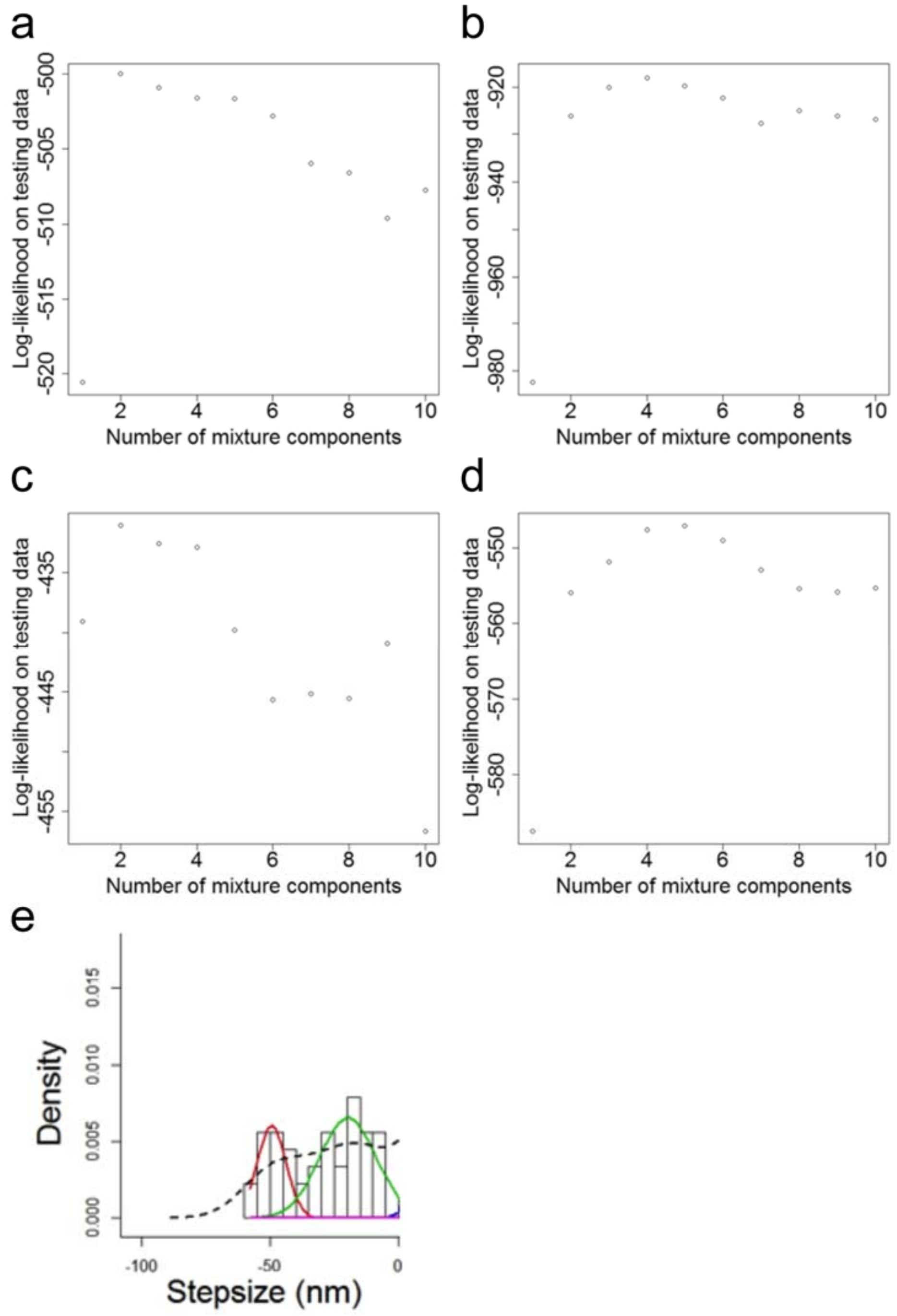

Supplementary Figure 5. Basis for fitting the Step Size Data. The plots shown in Figure 2 are the fitting results obtained by applying the normalMixEM procedure in the mixtools package in R[5]. The mixtools package is one of several available in R to fit mixture distributions or to solve model-based clustering. To minimize biased data fitting of our step size measurement data, we used a systematic and non-parametric analysis, instead of a manual approach to analyze the data. We fitted data with Gaussian mixture models with varying numbers of components with an expectation-maximization (EM) algorithm, using the mixtools package in R[5]. The EM algorithm implements an iterative method for finding maximum likelihood estimates of the parameters of the underlying distribution statistical models, where the model depends on unobserved latent variables. To avoid an arbitrary choice of the number of components in mixture models and the danger of under- or over-fitting the data, cross-validation was employed to select the number of components. Cross validation is a statistical model validation technique that, when performing a data model fitting, only **fits and builds the model to the part of the available data (a randomly chosen training set),** while reserving the rest of the available data as a **test set. The test set is then used to validate how well a given number of**

components can predict the unseen reserved data. The number of mixture components that generated the maximal log-likelihood of fitting the distribution of reserved data was adopted to fit step size mixture distributions of either full-length myosin X (**a**, **b**) or HMM (**c**, **d**) on either single F-actin filaments (**a**, **c**) or fascin-bundled F-actin filaments (**b, d**). These log likelihoods of a given number of components fitting the test data are depicted in panels **a**-**d**, above.
From this cross-validation testing, two components were chosen to fit single filament data (**a**, **c**), while four components were chosen to fit actin bundle data (**b**, **d**). Note however, that a five component model was a slightly better fit for (**d**) (HMM on bundles), due to two populations emerging from the backward steps. That fit is shown in panel (**e**). The values for the Gaussian components are -49 ± 6 (S.D) nm, -20 ± 11 (S.D) nm.

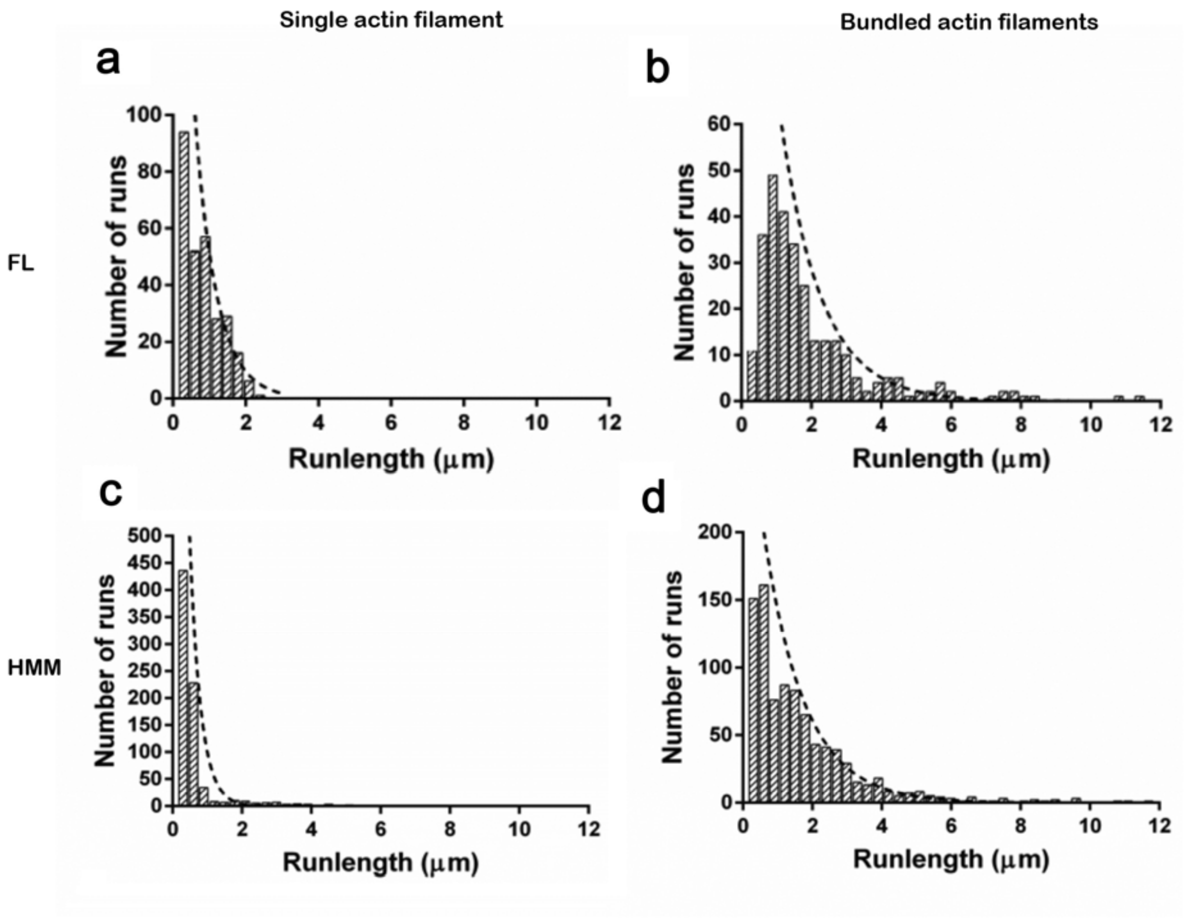

Supplementary Figure 6. Run lengths of myosin X dimers. Histograms of the run lengths of mWasabi-Myosin X full-length construct on single **(a)** and fascin-bundled **(b)** F-actin filaments are plotted. Histograms of the run lengths of Myosin X HMM on single **(c)** and fascin-bundled **(d)** F-actin filaments are plotted. The dashed curves in the histograms are the exponential fits, with the run length constant, λ, listed in Table 1.

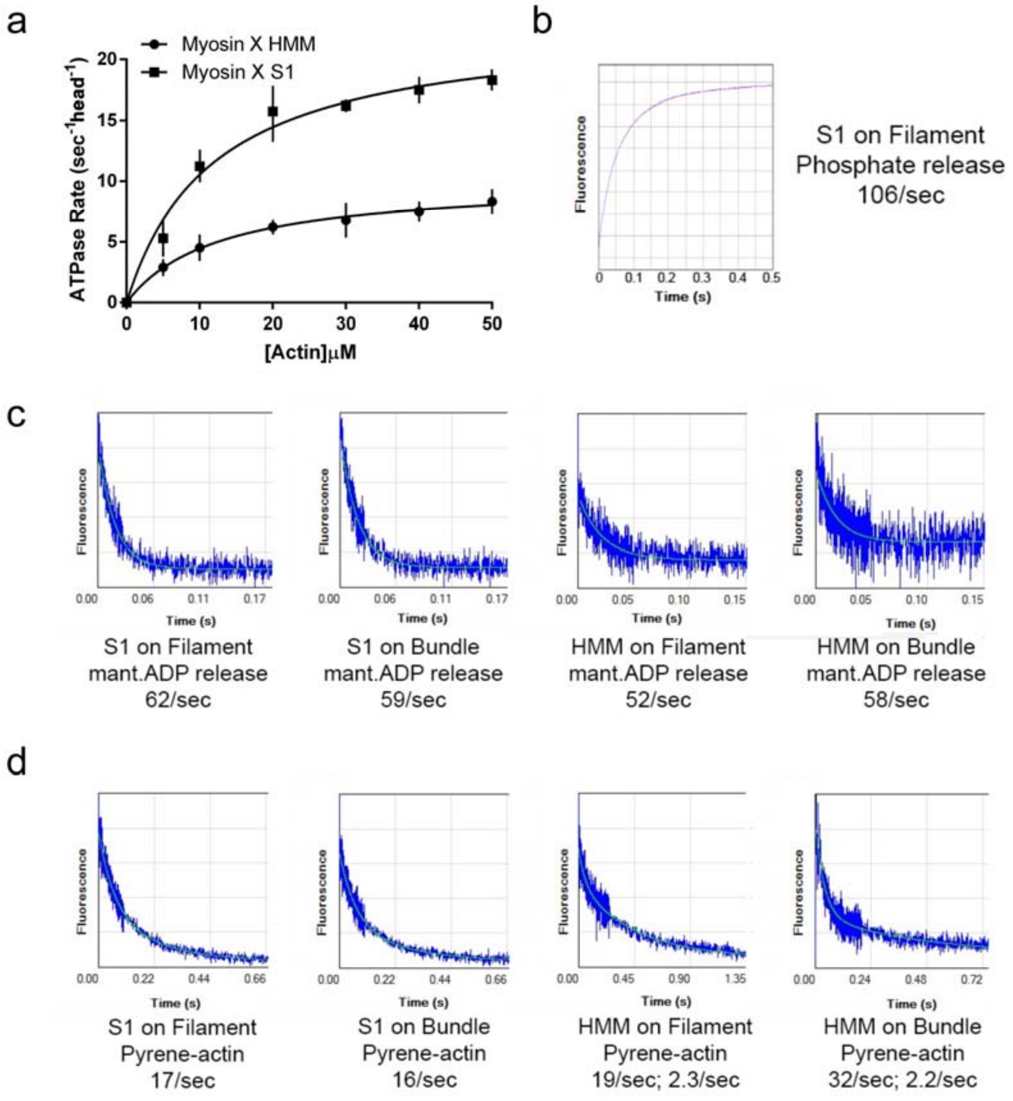

Supplementary Figure 7. Kinetics of myosin X on actin. (a) Actin-activated ATPase activity of myosin X. The actin concentration-dependence of the steady state ATPase activities ($sec^{-1}.head^{-1}$) of the zippered myosin X dimer (HMM) is shown with closed circles.The actin activated ATPase values of a single-headed (S1) myosin X monomer (truncated at the beginning of the coiled-coil) are shown in closed squares. In both cases, the values are an average of four preparations (±SD). The extrapolated maximal actin-activated ATPase for the myosin X HMM was 9.3 ± 2.3/sec (K_{ATPase} = 7.7μM), while the S1 was 19.6 ± 3.4/sec (K_{ATPase} = 9.6μM). **(b) Phosphate release from actin-myosin X.** Shown is a trace with a single exponential fit of phosphate release following the interaction of myosin X.ADP.Pi with 50μM actin. The average value (±SD) from multiple experiments (n=8) was 108 ± 4/sec. (**c) MantADP release from actin-myosin X.** Shown are traces from stopped flow experiments in which mantADP is competed off of the myosin X-actin complex (either S1 or HMM on single filaments or on bundles) by unlabeled ADP. The rate of change in fluorescence when the mantADP enters solvent was fit by a single exponential with the indicated rates. The average of the rates (±SD) from multiple experiments (n=9) are listed in Table 2. (**d) Rate of transition on pyrene-actin.** Shown are traces from stopped flow experiments in which myosin X (S1 or HMM with ADP.P_i bound) is allowed to bind to either single actin filaments or to actin bundles, which results in a change in the pyrene-actin fluorescence. The rate of change in fluorescence when the myosin binds was best fit by a single exponential for the S1 constructs, but was best fit by two exponentials for the HMM constructs, with the indicated rates. This implies that the transition is slowed on the lead head (gating), since the rear head cannot detach from actin. The average of the rates (±SD) from multiple experiments (n=9) are listed in Table 2.

a

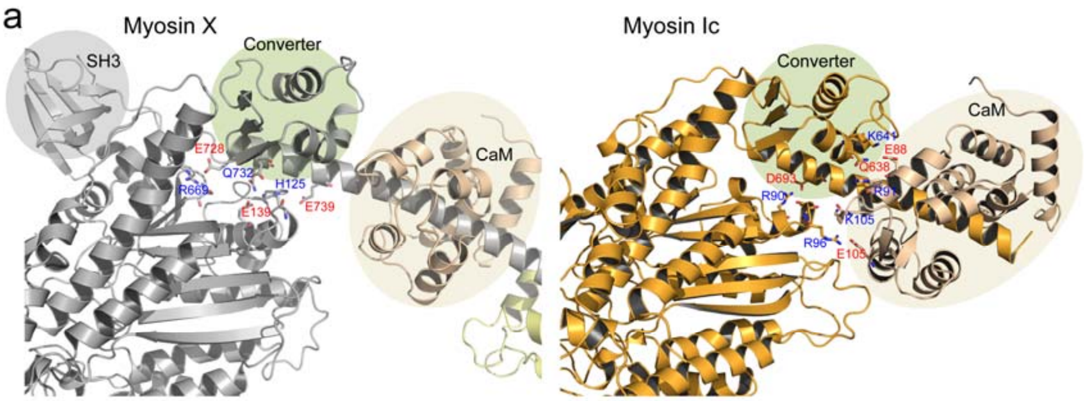

b

	Motor domain	Converter	Light Chain
Myosin X	Arg 669, S98, G97	Glu 728	------
	Lys 146	Glu731	------
	Glu139, L126	Gln732	------
	His 125	Glu 739	------
Myosin 1c (4BYF)	Arg 90	Asp 693	Ø
	Thr 94	------	Lys 95
	Arg96	------	Glu 15
	Ø	Gln 638	Arg 91, Glu 88
	Ø	Lys 641	Glu 88

c

Reference Myosin X (motor domain)	R.M.S.D
Myosin Vc	0.797
Myosin VI (2V26)	1.099
Myosin Ic (4BYF)	0.722
Smooth Muscle Myosin (1BR2)	0.881

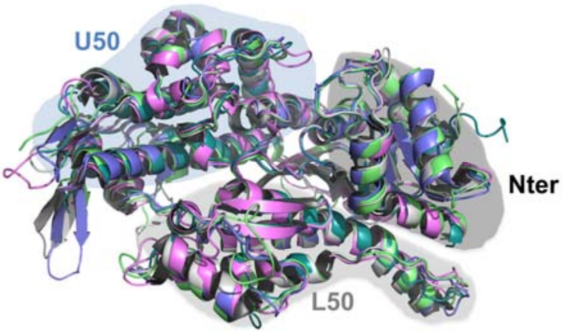

d

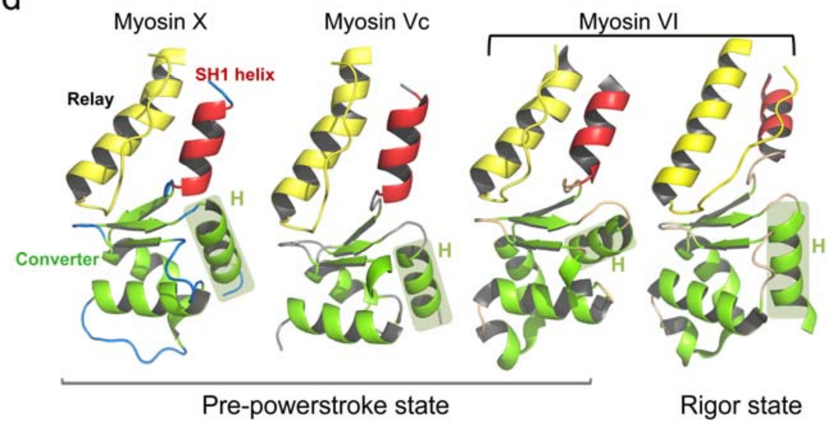

Supplementary Figure 8. The pre-powerstroke state of Myosin X. (a) Comparison of Myosin X and Myosin Ic pre-powerstroke state. Very different interactions are formed in the two myosin motors by the converter with the rest of the motor. The lever arm orientation differs drastically since the Myosin X converter mainly interacts with the Nter subdomain while in Myosin Ic, the interactions differ and involve the CaM light chain as well as the converter. **(b)** Table of the interactions that position the converter. **(c)** RMSD between the motor domain of myosin X and other myosins crystallized in the pre-powerstroke state. Note that the three main subdomains of the motor superimpose quite well. **(d)** The converter fold in myosin X in the pre-powerstroke state is similar to that of Myosin Vc, myosin II (not shown) and to the fold found for the Rigor state of Myosin VI as shown by the orientation of the beta-sheet and the last helix (H). It differs drastically from the fold of the pre-powerstroke Myosin VI converter[6], that undergoes a large conformational change during the powerstroke.

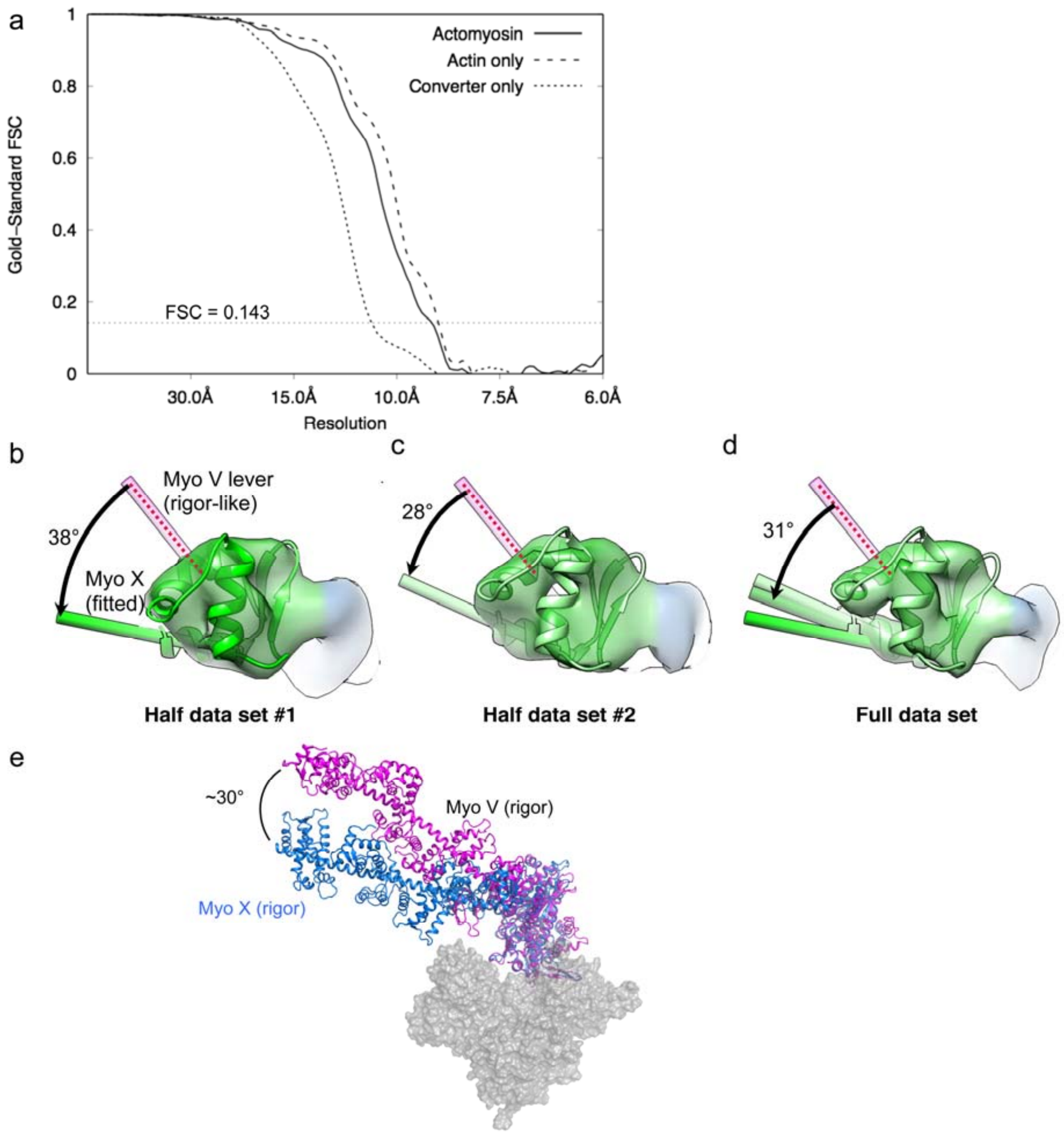

Supplementary Figure 9. Cryo-EM analysis of the myosin X rigor state on actin.
(a) Fourier Shell Correlation analysis of the cryo-EM reconstruction of myosin X-decorated actin filaments (rigor state). These estimates of the resolution were generated through the comparison of two independently refined and reconstructed 3D maps, representing equal halves of the data ('gold-standard' FSC; see Methods). **(b-d)** Modeling the converter/lever arm position from our nanometer-resolution cryo-EM maps of the myosin X rigor state on actin. These fits demonstrate the overall consistency of the rigid-body fits of this subdomain as performed in each of the three cryo-EM maps (individual half-data-sets as well as the full-data set reconstruction). The axis of the lever arm helix is also illustrated; for comparison, the axis of the myosin V lever arm helix is shown, along with measurements of angular difference between these two axes for each fitting. **(e)** Comparison of the Myosin X rigor state model docked on F-actin with that previously described for Myosin V in rigor[7].

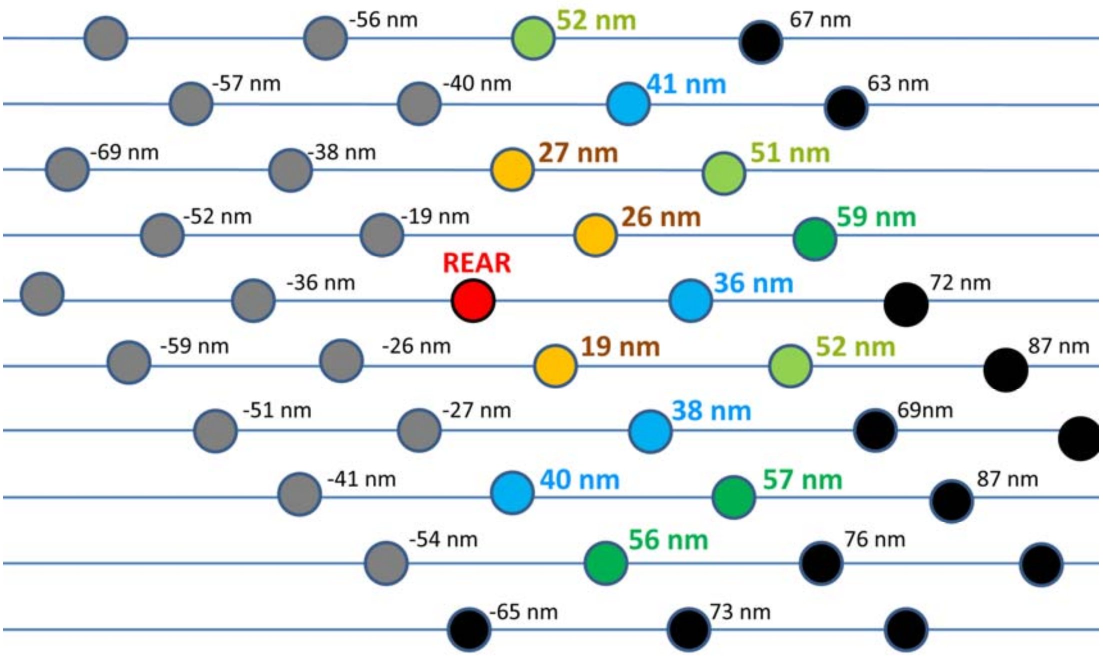

Supplementary Figure 10. Representation of actin hot spots in a fascin bundle. The blue lines represent parallel F-actin filaments with a hot spot for myosin binding found every 36 nm. The actin filaments are cross-linked by fascin and their hot spots are colored differently depending on the distance from the position of the rear head (marked in red).

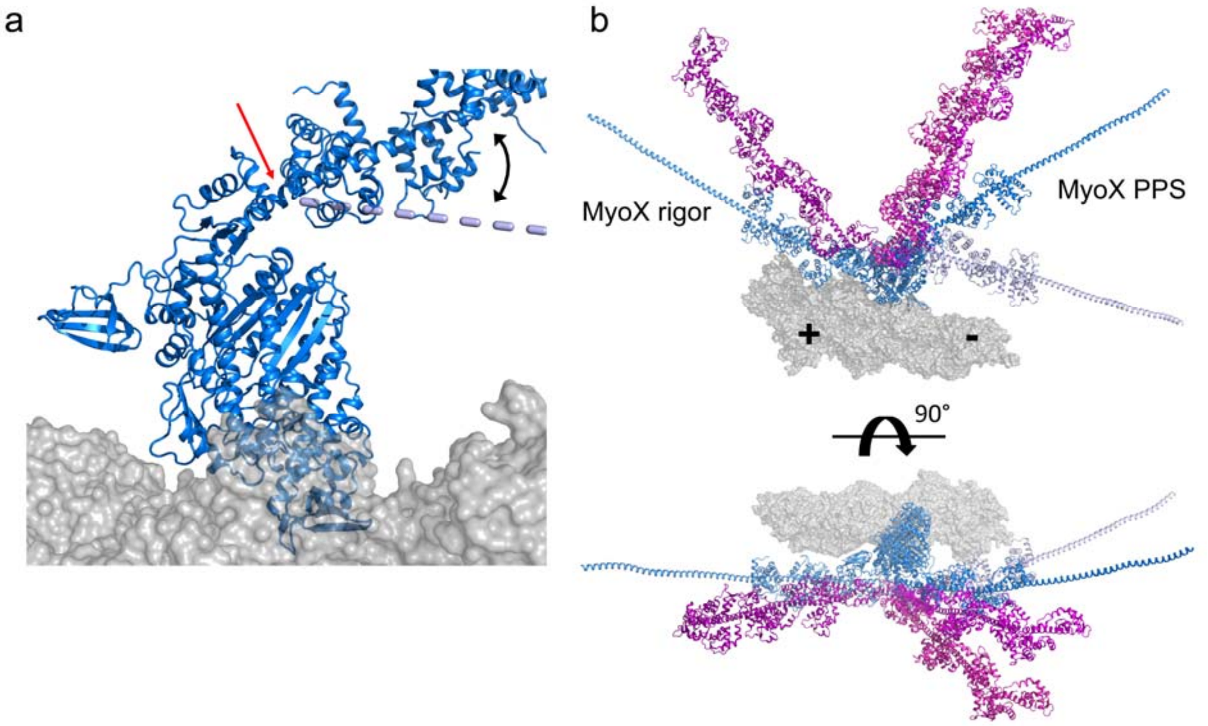

Supplementary Figure 11. Pliant region in myosins provides variation in the position of the lever arm. (a) The Myosin X MD2IQ PPS structure is docked on actin via the actin binding elements as described in Fig.4. The red arrow indicates the pliant region where bending of the heavy chain helix at the end of the converter can allow changes in the orientation of the rest of the lever arm (IQ motifs with LCs bound), see black arrow. The dashed line in grey indicates the orientation that the lever arm can adopt without much steric hindrance. **(b)** The powerstroke of myosin X (blue) and myosin V (magenta) is shown as in Fig.4. The position of the lever arm in grey represents the pre-powerstroke position when maximal bending at the pliant region occurs (without steric hindrance between the light chain and the motor domain). This indicates that the stroke can even be larger if the bending occurs at the pliant region.

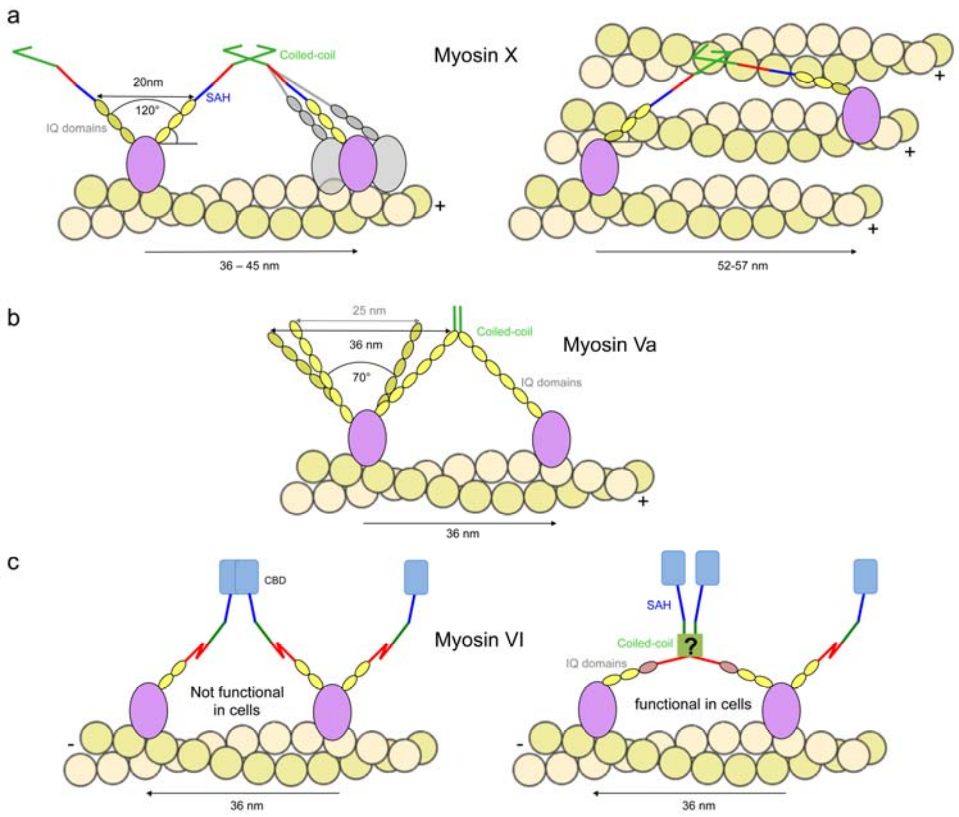

Supplementary Figure 12. Step sizes of unconventional myosins on F-actin. (a)-(c) Motor domains are in pink, the IQs/CaM region in yellow, the coiled-coil in green, the SAH region in blue and the helical region with hydrophobic residues in red **(a)** Scheme of the Myosin X stroke on F-actin as well as step sizes possible on a single filament (Left) and on actin bundles (Right). **(b)** Representation of the Myosin V 25 nm stroke and its 36 nm step size on F-actin. **(c)** Two models proposed for step sizes of Myosin VI on F-actin[8]. The model that dimerizes via the CBD is not functional in cells (Left) in contrast to the model containing a dimerization region prior to the SAH region (blue).

Myosin X shares a number of structural features and adaptations with the reverse motor, myosin VI, that creates distinct but highly specialized properties for these members of the superfamily. For instance, both motors display a broad distribution of step sizes, and a significant number of back steps in the absence of load that likely is due to their unusual lever arm structures. While both myosin VI and myosin X can weakly dimerize, a proximal dimerization region of myosin VI precedes the SAH (c), and that of myosin X follows the SAH (a). Note however that the definition of the myosin VI lever arm and its ability to function as a monomer has been controversial[6,8-11]. Myosin X, like myosin VI, appears to exist primarily as folded monomers in cells[12-13] that are capable of regulated dimerization when two monomers interact with their cargoes[6]. As is the case for myosin VI[8], proper dimerization of myosin X is essential for its function, allowing its movement to the tip of the filopodia and its proper localization in cells[14-15].

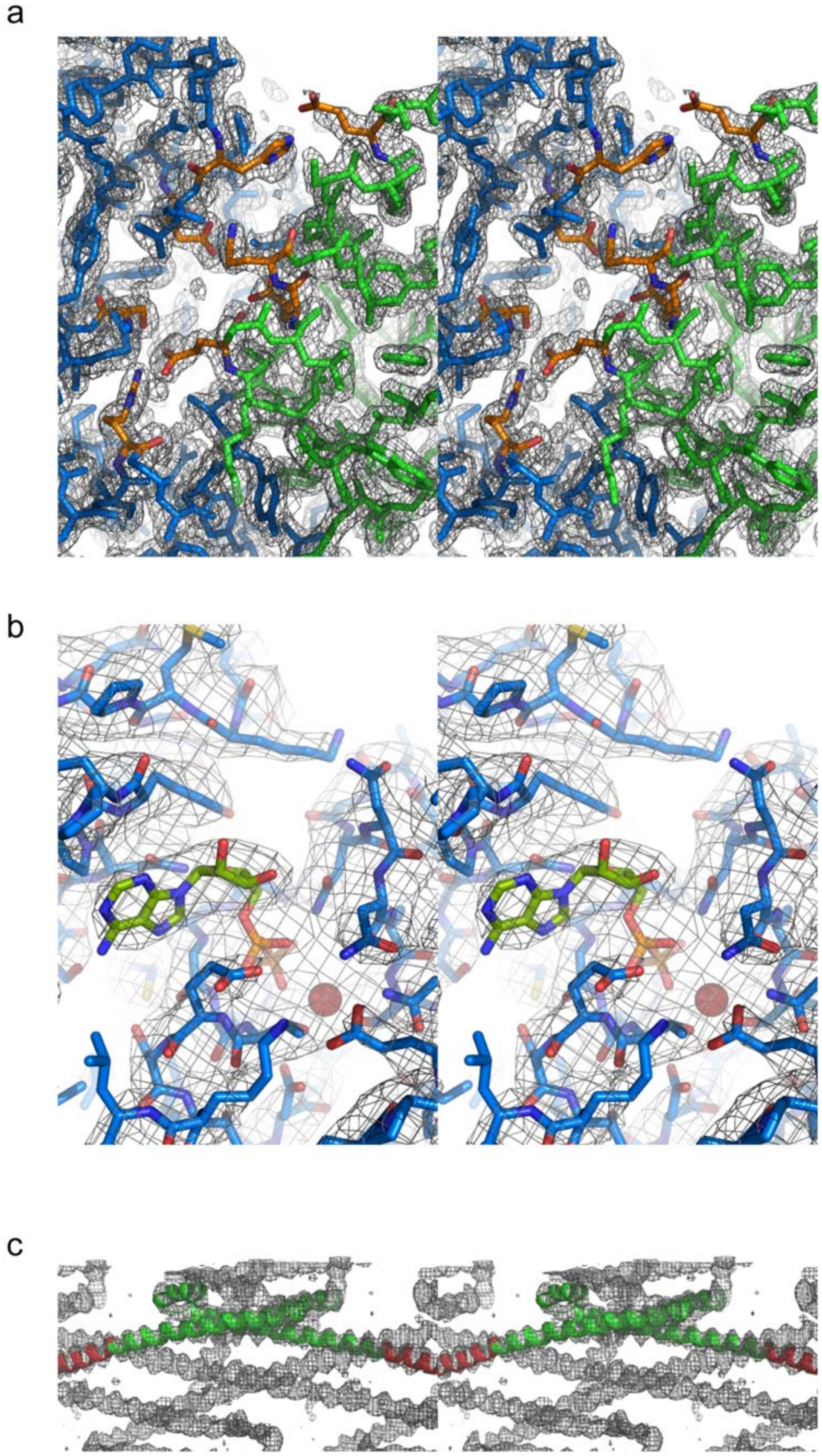

Supplementary Figure 13. Stereoviews of MyoX structures. (**a**) Sticks representation of MyoX MD construct contoured at 2.0 sigma and level 1.0 (gray mesh, Pymol). The orange patch correspond to residues implicated in interaction between converter (green) and the N-terminal region of MD (blue) (**b**) Sticks representation of nucleotide binding pocket of MyoX MD-2IQ construct. Map in mesh representation (gray) at 2.0 sigma and contour level 1.0. (**c**) Cartoon representation of IQ3-SAH-CC with map at 2.0 sigma and contour level 1.5.

Supplementary Table 1. Comparison of different chimeras of Myosin X with the Full-length motor.

	M5	M10 short chimera	M10-M5 chimera	M10- short Lzip chimera	M10 full length / unrestricted HMM
**Data publications**	Bao et al Plos One 2013[17]	Nagy et al Pnas 2008[19]<br>Ricca et al Biophys J 2010[20]	Sun et al NSMB 2010[16]<br>Takagi et al PNAS 2014[18]	Bao et al Plos One 2013[17]	This paper : Ropars et al.
**Construct**	**HMM (6IQ-cc)**	**1-920+Lzip**	**1-938+Myo5 cc**	**1-940+Lzip ; 1-936+Lzip**	**Full Length / 1-938+19aa linker+Lzip**
**On single actin**<br>Velocity (nm/s)<br>Processivity<br>Run length (nm)	 420<br>yes<br>1221	 330 (gliding assay)<br>No, supercoils actin<br>170	 210<br>yes<br>950 (suspended actin)	 190<br>yes<br>755	 312<br>yes<br>810
**On Fascin-actin bundles**<br>Velocity (nm/s)<br>Processivity<br>Run length (nm)	 430<br>yes<br>1255	 240<br>yes<br>630	 167<br>yes<br>1160	 165<br>yes<br>1465	 **660 ***<br>yes<br>1950
**Selectivity on bundles**	no	Processive only on bundle	no	Yes, in run length, no in velocity	Yes, with faster and longer runs on bundle
**Steps (nm)**<br>Single actin filaments<br>Actin bundle	 36 nm<br>36 nm, few 22 nm	 None<br>18 /-18 nm	 34 nm-left handed path, single<br>28 nm, some -17 nm more variable	 31 nm[19], ~36 nm few -36 nm [18]<br>31 and 21 nm	 36 nm<br>18 nm, 38 nm, and 52-57 nm
**Dimerisation**	Parallel coiled-coil	Unknown	Likely conflict between native anti-parallel CC fused to a strong parallel CC, *From EM studies[16], Head-head angle is ~120° (like myo5) rather than 180°*		Anti-parallel, *Knight et al.[21] observed ~180° angle in Myosin X*
**Lever arm length** (predicted)	26 nm (Conv-6IQ)<br>Heads are gated	Short	Lever max. : 19 nm (Conv-3IQ-SAH)<br>No gaiting		Lever max. : 24 nm<br>Heads are gated
Stroke (measured, calculated)	25 nm	Unknown	17 nm		(120° swing + 24 nm) ~ 41nm
**Conclusion**	Head with long 26 nm rigid lever arm + parallel coiled-coil restrain the angle to < 120°	Likely zipping helical region and produces a chimera with short lever arm and head constrained	Similar M10-short Lzip chimera<br><br>Native dimerization not preserved due to close fusion to parallel coiled-coil	Likely zippering the helical region to constrain the lever/ lever angle <120° : distance head/head shorter	Antiparallel coiled-coil separates the heads. Long and semi-flexible lever arm (coiled-coil is part of it). This allows a flat conformation – preference for 180°between heads.
**Distance max.** reachable for HMM on actin	45 nm	18-20 nm	36 nm		52-57 nm (without need of SAH unfolding)

**** similar to velocity in vivo in filopodia[22] 580 and 840 nm/s at 25°C and 37°C respectively***

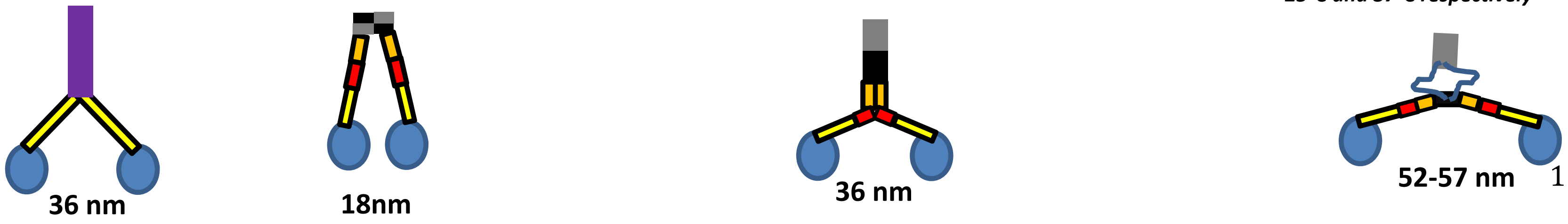

Supplementary Table 2. Crystallography statistics

	MyoX MD (5I0H)	MyoX MD-2IQ (5I0I)	Myosin MD Vc (5HMP)	MyoX IQ3-SAH-CC (5HMO)
**Data collection**				
Space group	P1	$P2_12_12_1$	$P2_1$	$P4_32_12$
Cell dimensions				
a,b,c (Å)	76.48, 78.30, 78.67	113.95, 173.41, 178.75	103.78, 66.74, 131.26	69.71, 69.71, 236.42
α,β,γ (°)	75.69, 86.38, 76.12	90, 90, 90	90, 104.45, 90	90, 90, 90
Resolution (Å)	50.0-1.80 (1.864-1.8)	50.0-3.15 (3.23-3.15)	48.5-2.39 (2.54-2.39)	49.37-3.49 (3.70-3.49)
R_{sym} or R_{merge}	5.2 (65.1)	16.3 (252.1)	4.9(61.2)	10.76 (181.9)
$I/\sigma I$	15.96 (2.07)	14.48 (1.15)	13.47 (1.85)	15.74 (2.02)
Completeness (%)	97.4 (95.3)	99.9 (99.7)	98.3 (96.4)	99.8 (98.9)
Redundancy	4.8 (3.98)	16.9 (16.81)	3 (2.9)	13.7 (2.08)
**Refinement**				
Resolution (Å)	26.35-1.80 (1.85-1.80)	49.53-3.15 (3.23-3.15)	48.52-2.39 (2.43-2.39)	22.85-3.49 (3.90-3.49)
No. of reflections	155150	61853	67573	7979
R_{work} / R_{free}	20.17/24.71 (24.71-20.39)	19.30/21.85 (29.10-25.73)	20.5/22.7 (22.4-20.1)	30.73/34.78 (36.07-32.92)
No. atoms				
Protein	11854	15203	11073	1946
Ligand/ion	161	132	82	-
Water	1258	33	377	-
B-factors				
Protein	36.19	126.33	82.58	193.05
Ligand/ion	44.46	132.76	58.69	-
Water	44.85	83.27	65.43	-
R.m.s deviations				
Bond lengths (Å)	0.010	0.010	0.003	0.010
Bond angles (°)	1.000	1.22	0.638	1.41
Favoured / allowed/oulier Ramachandran angles	97%, 2.7% and 0.41%	93%, 5.2% and 2.3%	97%, 3.1% and 0.22%	92.8%, 7.2% and 0%,

*Highest resolution shell is shown in parenthesis.

Supplementary Note relative to the previous myosin X chimeras (Supplementary Table 1)

Comparison of the motility data and step size reports about myosin X.

As shown in Supplementary Table 1, major differences have been reported for different myosin X artificial dimers[16-20]. We attribute the discrepancy in our results with those of other reports[16-20] to the manner in which the zippered dimers were created. From our study of the native anti-parallel dimerization region, the placement of either the leucine zipper, GCN4[17-19] or the coiled-coil of myosin V[16] would have disrupted the normal dimerization of myosin X (Supplementary Table 1, Supplementary Fig.12). While Tagaki et al.[18] suggested that there were a sufficient number of amino-acids between the leucine zipper and dimerization region to preserve native dimerization, our analysis suggests that a larger linker, such as we engineered, would be necessary to avoid disrupting the anti-parallel coiled-coil and thus the native dimerization motif of myosin X (Supplementary Fig.12). In two published chimeras[16,18], twenty additional residues of the native sequence were present as compared to the first chimera of Nagy et al[19]. This apparently leads to a dimeric molecule that prefers 36nm steps, and is thus well suited to stepping on single actin filaments, on which the hot spots are distant by 36nm (Fig.5d). This is in contrast to the short, 18nm steps that the more truncated construct[19-20] generates. This short step size is not well suited to single filaments, as the corresponding actin binding site for the lead head is on the side of the filament (Fig.5d), artificially creating a preferential stepping on bundles. This construct[19] lacked 15 residues of the dimerization region and was directly fused to a GCN4 leucine zipper. How this chimera dimerizes is unclear, but this dimerization must somehow shorten the lever arm and/or constrain the heads resulting in a short step size despite the presence of the native lever arm (Supplementary Table 1).

Our observations as well as earlier published results[13,15] suggest the affinity of the myosin X anti-parallel coiled coil to be in the µM range. Thus given that the effective concentration will be much higher than µM in the zippered HMM constructs, it is highly unlikely that the coiled coil is not formed in the presence of the leucine zipper. We would not expect to see gating if a long flexible linker (followed by the leucine zipper) loosely tethered the two heads. While the reported size of the largest steps for the zippered HMM (57 ± 2 nm) was slightly larger than that of the full-length dimer (52 ± 5 nm), the difference was not statistically significant. However, the trend could indicate that additional residues C-terminal to the coiled coil that were removed from the zippered dimer can help stabilize the anti-

parallel coiled-coiled. This may occasionally allow the coiled coil to partially unzip in the zippered dimers, generating slightly larger steps than in the full-length molecule.

Supplementary References :

1. Kerber, M. L. & Cheney, R. E. Myosin-X: a MyTH-FERM myosin at the tips of filopodia. J. Cell. Sci. 124, 3733-3741 (2011).
2. Umeki, N. et al. Phospholipid-dependent regulation of the motor activity of myosin X. Nat. Struct. Mol. Biol. 18, 783-788 (2011).
3. Weber, K. L., Sokac, A. M., Berg, J. S., Cheney, R. E. & Bement, W. M. A microtubule-binding myosin required for nuclear anchoring and spindle assembly. Nature 431, 325-329 (2004).
4. Zhang, H. et al. Myosin-X provides a motor-based link between integrins and the cytoskeleton. Nat. Cell Biol. 6, 523-531 (2004).
5. Benaglia,T., Chaurveau,D., Hunter, D.R., Young, D. mixtools: An R Package for analyzing Mixture Models. J Stat Softw. 32, 1-29 (2009).
6. Sweeney, H. L. & Houdusse, A. Myosin VI rewrites the rules for myosin motors. Cell 141, 573-582 (2010).
7. Wulf, S. et al. Force producing ADP state of Myosin bound to Actin. Proc Natl Acad Sci USA 113, E1844-52 (2016).
8. Mukherjea, M. et al. Myosin VI must dimerize and deploy its unusual lever arm in order to perform its cellular roles. Cell Rep. 8, 1522-1532 (2014).
9. Spink, B. J., Sivaramakrishnan, S., Lipfert, J., Doniach, S. & Spudich, J. A. Long single alpha-helical tail domains bridge the gap between structure and function of myosin VI. Nat. Struct. Mol. Biol. 15, 591–597 (2008).
10. Mukherjea, M. et al. Myosin VI dimerization triggers an unfolding of a three-helix bundle in order to extend its reach. Mol. Cell 35, 305-315 (2009).
11. Bond, L. M., Arden, S. D., Kendrick-Jones, J., Buss, F. & Sellers, J. R. Dynamic exchange of myosin VI on endocytic structures. J. Biol. Chem. 287, 38637–38646 (2012).
12. Lister, I. et al. A monomeric myosin VI with a large working stroke. EMBO J. 23, 1729-1738 (2004).
13. Umeki, N. et al. Phospholipid-dependent regulation of the motor activity of myosin X. Nat. Struct. Mol. Biol. 18, 783-788 (2011).
14. Tokuo, H., Mabuchi, K. & Ikebe, M. The motor activity of myosin-X promotes actin fiber convergence at the cell periphery to initiate filopodia formation. J. Cell Biol. 179, 229-238 (2007).
15. Watanabe, T. M., Tokuo, H., Gonda, K., Higuchi, H. & Ikebe, M. Myosin-X induces filopodia by multiple elongation mechanism. J. Biol. Chem. 285, 19605-19614 (2010).
16. Sun, Y. *et al.* Single-molecule stepping and structural dynamics of myosin X. *Nat. Struct. Mol. Biol.* **17,** 485-91 (2010).
17. Bao, J., Huck, D., Gunther, L. K., Sellers, J. R. & Sakamoto, T. Actin structure-dependent stepping of myosin 5a and 10 during processive movement. *PLoS ONE* **8,** e74936 (2013).
18. Takagi, Y. *et al.* Myosin-10 produces its power-stroke in two phases and moves processively along a single actin filament under low load. *Proc. Natl. Acad. Sci. U.S.A.* **111,** E1833-42 (2014).
19. Nagy, S. *et al.* A myosin motor that selects bundled actin for motility. *Proc. Natl. Acad. Sci. U.S.A.* **105,** 9616-20 (2008).
20. Ricca, B. L. & Rock, R. S. The stepping pattern of myosin X is adapted for processive motility on bundled actin. *Biophys. J.* **99,** 1818-26 (2010).
21. Knight, P. J. et al. The predicted coiled-coil domain of myosin 10 forms a novel elongated domain that lengthens the head. J. Biol. Chem. 280, 34702-8 (2005).
22. Kerber, M. L. et al. A novel form of motility in filopodia revealed by imaging myosin-X at the single-molecule level. Curr. Biol. 19, 967-73 (2009).
